# Supplementary material for: Role of oral health in heart and vascular health: A population-based study
Source: PLoS One. 2024 Apr 18;19(4):e0301466. doi: 10.1371/journal.pone.0301466 (PMC11025934; doi:10.1371/journal.pone.0301466)
Supplement: S1 Table — (DOCX) [file pone.0301466.s001.docx]

| **S1 Table. Bootstrap analysis of oral health indicators and their association with cardiovascular outcomes** | | | | | | | | | |
| --- | --- | --- | --- | --- | --- | --- | --- | --- | --- |
| **Characteristics** | | **Hypertension** | | **Cardiovascular disease** | | **Hypercholesterolaemia** | | **Cardiovasular risk profile** | |
|  |  | Boostrap OR (95% CI) | P value | Boostrap OR (95% CI) | P value | Boostrap OR (95% CI) | P value | Boostrap OR (95% CI) | P value |
| **Gender** | Male |  |  |  |  |  |  |  |  |
|  | Female | 1.18 [0.99-1.40] | 0.062 | 0.95 [0.77-1.16] | 0.6 | 1.16 [0.95-1.41] | 0.146 | 1.11 [0.95-1.29] | 0.199 |
| **Age groups** | 65+ |  |  |  |  |  |  |  |  |
|  | 15-34 | 0.13 [0.08-0.21] | <0.001 | 0.40 [0.23-0.67] | 0.001 | 0.25 [0.14-0.47] | <0.001 | 0.11 [0.08-0.16] | <0.001 |
|  | 35-64 | 0.45 [0.35-0.56] | <0.001 | 0.62 [0.48-0.80] | <0.001 | 0.66 [0.50-0.86] | 0.003 | 0.41 [0.32-0.51] | <0.001 |
| **Area of residence** | Rural |  |  |  |  |  |  |  |  |
|  | Urban |  |  |  |  | 1.26 [1.02-1.55] | 0.034 | 1.11 [0.95-1.31] | 0.191 |
| **Educational attainment** | Primary |  |  |  |  |  |  |  |  |
|  | Secondary | 0.71 [0.59-0.87] | 0.001 | 0.87 [0.69-1.10] | 0.242 | 1.09 [0.86-1.37] | 0.475 | 0.85 [0.70-1.03] | 0.099 |
|  | Tertiary | 0.63 [0.49-0.81] | <0.001 | 1.23 [0.93-1.62] | 0.148 | 1.42 [1.06-1.90] | 0.018 | 1.00 [0.78-1.28] | 0.987 |
| **Employment** | Unemployed |  |  |  |  |  |  |  |  |
|  | Employed | 0.89 [0.72-1.12] | 0.324 | 0.68 [0.52-0.88] | 0.004 | 1.12 [0.84-1.48] | 0.443 | 0.92 [0.76-1.13] | 0.44 |
| **Financial status** | Average |  |  |  |  |  |  |  |  |
|  | Good | 0.90 [0.75-1.09] | 0.285 | 1.16 [0.91-1.48] | 0.219 | 0.93 [0.73-1.17] | 0.524 |  |  |
|  | Bad | 0.95 [0.73-1.24] | 0.71 | 1.12 [0.86-1.46] | 0.407 | 1.12 [0.85-1.50] | 0.417 |  |  |
| **Income quintiles** | First |  |  |  |  |  |  |  |  |
|  | Second | 0.87 [0.68-1.10] | 0.239 |  |  | 1.08 [0.81-1.43] | 0.601 | 0.92 [0.74-1.16] | 0.493 |
|  | Third | 0.76 [0.59-0.98] | 0.037 |  |  | 0.89 [0.65-1.22] | 0.473 | 0.88 [0.69-1.11] | 0.273 |
|  | Fourth | 0.96 [0.74-1.26] | 0.776 |  |  | 1.23 [0.89-1.69] | 0.213 | 1.08 [0.84-1.38] | 0.569 |
|  | Fifth | 0.82 [0.59-1.14] | 0.232 |  |  | 1.13 [0.77-1.68] | 0.53 | 0.83 [0.61-1.12] | 0.225 |
| **Smoking** | Smoker |  |  |  |  |  |  |  |  |
|  | Non-smoker | 1.13 [0.93-1.36] | 0.211 |  |  | 1.12 [0.89-1.43] | 0.335 |  |  |
| **BMI** | Overweight and Obese |  |  |  |  |  |  |  |  |
|  | Normal | 0.43 [0.36-0.52] | <0.001 |  |  | 0.55 [0.44-0.68] | <0.001 | 0.49 [0.41-0.57] | <0.001 |
| **Alcohol use** | Drinker |  |  |  |  |  |  |  |  |
|  | Non-drinker | 0.77 [0.64-0.93] | 0.007 | 1.06 [0.86-1.30] | 0.597 | 0.82 [0.67-1.02] | 0.077 |  |  |
| **Has Chronic disease** | No |  |  |  |  |  |  |  |  |
|  | Yes | 5.67 [4.67-6.89] | <0.001 | 4.60 [3.45-6.14] | <0.001 | 3.24 [2.50-4.18] | <0.001 | 5.55 [4.68-6.58] | <0.001 |
| **Self-perceived health** | Average |  |  |  |  |  |  |  |  |
|  | Good | 0.75 [0.63-0.91] | 0.003 | 0.47 [0.36-0.60] | <0.001 | 0.60 [0.47-0.76] | <0.001 | 0.55 [0.46-0.66] | <0.001 |
|  | Bad | 1.13 [0.87-1.45] | 0.363 | 2.49 [1.95-3.17] | <0.001 | 1.66 [1.29-2.13] | <0.001 | 1.57 [1.19-2.08] | 0.002 |
| **Self-perceived oral health** | Average |  |  |  |  |  |  |  |  |
|  | Good | 0.84 [0.69-1.02] | 0.074 | 1.07 [0.84-1.35] | 0.592 | 1.04 [0.83-1.29] | 0.742 | 0.92 [0.77-1.10] | 0.364 |
|  | Bad | 0.87 [0.70-1.08] | 0.207 | 1.18 [0.94-1.48] | 0.158 | 1.21 [0.94-1.55] | 0.141 | 1.18 [0.94-1.47] | 0.155 |
| **Number of permanent teeth extracted due to decay, not replaced** | None |  |  |  |  |  |  |  |  |
|  | 1 to 5 | 1.36 [0.85-2.17] | 0.206 | 1.01 [0.55-1.88] | 0.965 | 1.34 [0.75-2.41] | 0.327 |  |  |
|  | 6 to 19 | 2.05 [1.25-3.37] | 0.005 | 1.35 [0.73-2.51] | 0.334 | 1.26 [0.69-2.29] | 0.453 |  |  |
|  | More than 20 | 1.57 [0.93-2.65] | 0.089 | 1.26 [0.67-2.35] | 0.475 | 1.19 [0.65-2.20] | 0.57 |  |  |
| **Has filled teeth** | No |  |  |  |  |  |  |  |  |
|  | Yes |  |  | 0.87 [0.69-1.09] | 0.216 |  |  | 1.06 [0.88-1.28] | 0.554 |
| **Presence of active caries** | No |  |  |  |  |  |  |  |  |
|  | Yes |  |  | 1.19 [0.95-1.50] | 0.138 |  |  | 0.98 [0.81-1.17] | 0.792 |
| **Has mobile teeth** | No |  |  |  |  |  |  |  |  |
|  | Yes | 1.07 [0.79-1.43] | 0.672 | 1.00 [0.74-1.35] | 0.984 | 1.34 [0.99-1.82] | 0.054 | 1.40 [1.03-1.89] | 0.031 |
| **Has gum bleeding when brushing teeth** | No |  |  |  |  |  |  |  |  |
|  | Yes | 0.97 [0.77-1.22] | 0.793 | 1.66 [1.29-2.15] | <0.001 | 1.43 [1.11-1.82] | 0.005 |  |  |
| **Has permanent teeth missing due to decay (extracted) and not replaced** | No |  |  |  |  |  |  |  |  |
|  | Yes | 0.90 [0.74-1.09] | 0.267 | 0.98 [0.80-1.22] | 0.881 | 1.05 [0.84-1.31] | 0.662 | 0.92 [0.77-1.10] | 0.373 |
| **Oral health** | Optimal |  |  |  |  |  |  |  |  |
|  | Suboptimal |  |  | 1.26 [0.74-2.13] | 0.394 |  |  | 1.17 [0.90-1.54] | 0.247 |
| **Presence of prosthetic tooth replacement** | No |  |  |  |  |  |  |  |  |
|  | Yes | 1.39 [1.17-1.66] | <0.001 | 1.32 [1.07-1.64] | 0.011 | 1.55 [1.24-1.95] | <0.001 | 1.70 [1.43-2.02] | <0.001 |
| **Last dental Checkup** | More than a year ago |  |  |  |  |  |  |  |  |
|  | Less than 6 months ago | 1.02 [0.84-1.25] | 0.822 | 1.12 [0.88-1.42] | 0.363 | 1.52 [1.21-1.92] | <0.001 | 1.31 [1.08-1.59] | 0.006 |
|  | less than a year but more than 6 months ago | 0.94 [0.75-1.18] | 0.573 | 0.88 [0.67-1.16] | 0.357 | 1.12 [0.87-1.44] | 0.379 | 0.91 [0.73-1.12] | 0.361 |

CVDs: cardiovascular diseases; BMI: body mass index; Bootstrap odds ratios (ORs) with 95% confidence intervals (CIs) for the association between various characteristics, including oral health indicators, and the prevalence of hypertension, cardiovascular disease, hypercholesterolemia, and a combined cardiovascular risk profile. Bootstrap results are based on 1,000 replications. ORs are adjusted for other covariates as specified in the model. Variables that were discarded with elastic net regularization are shaded with grey.
