# Supplementary material for: Role of oral health in heart and vascular health: A population-based study
Source: PLoS One. 2024 Apr 18;19(4):e0301466. doi: 10.1371/journal.pone.0301466 (PMC11025934; doi:10.1371/journal.pone.0301466)
Supplement: S2 Table — (DOCX) [file pone.0301466.s002.docx]

| **S2 Table. Elastic net selection results with α=0.5: predictors for hypertension and cardiovascular diseases** | | | | |
| --- | --- | --- | --- | --- |
| **Predictor variable** | **Hypertension (Elastic Net)** | **Hypertension (Post-est OLS)** | **Cardiovascular diseases (Elastic Net)** | **Cardiovascular diseases (Post-est OLS)** |
|  |  |  |  |  |
| Age | -0.0524 | -0.0536 | -0.0369 | -0.0412 |
| Gender | 0.0168 | 0.0243 | ------------------ | ------------------ |
| Residence | ------------------ | ------------------ | ------------------ | ------------------ |
| Education | -0.0401 | -0.0436 | -0.0002 | -0.0025 |
| Employment | -0.0504 | -0.0500 | -0.0621 | -0.0605 |
| Financial status | -0.0040 | -0.0071 | 0.0083 | 0.0140 |
| Income | -0.0043 | -0.0058 | ------------------ | ------------------ |
| BMI | -0.1428 | -0.1470 | ------------------ | ------------------ |
| Smoking | 0.0335 | 0.0392 | ------------------ | ------------------ |
| Alcohol | -0.0217 | -0.0328 | 0.0203 | 0.0260 |
| Self-perceived health | -0.0023 | -0.0044 | 0.0638 | 0.0704 |
| Self-perceived oral health | -0.0089 | -0.0124 | 0.0112 | 0.0150 |
| Presence of chronic diseases | 0.3674 | 0.3708 | 0.2179 | 0.2232 |
| Number of teeth extracted | 0.0523 | 0.0525 | 0.0386 | 0.0357 |
| Has filled teeth | ------------------ | ------------------ | -0.0078 | -0.0161 |
| Presence of active caries | ------------------ | ------------------ | 0.0032 | 0.0128 |
| Has mobile teeth | 0.0236 | 0.0353 | 0.0196 | 0.0261 |
| Gum bleeding | -0.0125 | -0.0217 | 0.0548 | 0.0651 |
| Teeth extracted not replaced | 0.0164 | 0.0224 | 0.0007 | 0.0103 |
| Oral health |  |  | 0.0048 | 0.0242 |
| Has prosthetically replaced teeth | 0.0813 | 0.0834 | 0.0172 | 0.0355 |
| Last dental checkup | -0.0070 | -0.0094 | -0.0035 | -0.0065 |
