# Supplementary material for: Role of oral health in heart and vascular health: A population-based study
Source: PLoS One. 2024 Apr 18;19(4):e0301466. doi: 10.1371/journal.pone.0301466 (PMC11025934; doi:10.1371/journal.pone.0301466)
Supplement: S3 Table — (DOCX) [file pone.0301466.s003.docx]

| **S3 Table. Elastic net selection results with α=0.5: predictors for hypercholesterolaemia and cardiovascular risk profile** | | | | |
| --- | --- | --- | --- | --- |
| **Predictor variable** | **Hypercholesterolaemia (Elastic Net)** | **Hypercholesterolaemia (Post-est OLS)** | **Cardiovascular risk profile (Elastic Net)** | **Cardiovascular risk profile (Post-est OLS)** |
|  |  |  |  |  |
| Age | -0.02126 | -0.02324 | -0.04805 | -0.04909 |
| Gender | 0.012564 | 0.019216 | 0.008067 | 0.013914 |
| Residence | 0.02095 | 0.024027 | 0.004081 | 0.013169 |
| Education | 0.015014 | 0.016629 | -0.00759 | -0.01087 |
| Employment | -0.0046 | -0.0069 | -0.05515 | -0.05317 |
| Financial status | 0.00469 | 0.008198 | ------------------ | ------------------ |
| Income | 0.000654 | 0.002611 | -0.00332 | -0.00494 |
| BMI | -0.06458 | -0.06981 | -0.12655 | -0.13054 |
| Smoking | 0.011102 | 0.014192 | ------------------ | ------------------ |
| Alcohol | -0.0027 | -0.01138 | ------------------ | ------------------ |
| Self-perceived health | 0.015377 | 0.019633 | -0.00408 | -0.00874 |
| Self-perceived oral health | 0.010212 | 0.012937 | 0.014465 | 0.018985 |
| Presence of chronic diseases | 0.158351 | 0.161082 | 0.419587 | 0.420987 |
| Number of teeth extracted | 0.005269 | 0.007988 | ------------------ | ------------------ |
| Has filled teeth | ------------------ | ------------------ | -0.01713 | -0.01958 |
| Presence of active caries | ------------------ | ------------------ | -0.01765 | -0.02752 |
| Has mobile teeth | 0.054705 | 0.061232 | 0.084244 | 0.094042 |
| Gum bleeding | 0.04603 | 0.051682 | ------------------ | ------------------ |
| Teeth extracted not replaced | 0.010527 | 0.018315 | 0.019532 | 0.024084 |
| Oral health | ------------------ | ------------------ | 0.065189 | 0.070365 |
| Has prosthetically replaced teeth | 0.054794 | 0.057693 | 0.159435 | 0.16038 |
| Last dental checkup | 0.006377 | 0.010025 | -0.01574 | -0.01852 |
