# Supplementary figures and images for: Role of oral health in heart and vascular health: A population-based study
Source: PLoS One. 2024 Apr 18;19(4):e0301466. doi: 10.1371/journal.pone.0301466 (PMC11025934; doi:10.1371/journal.pone.0301466)

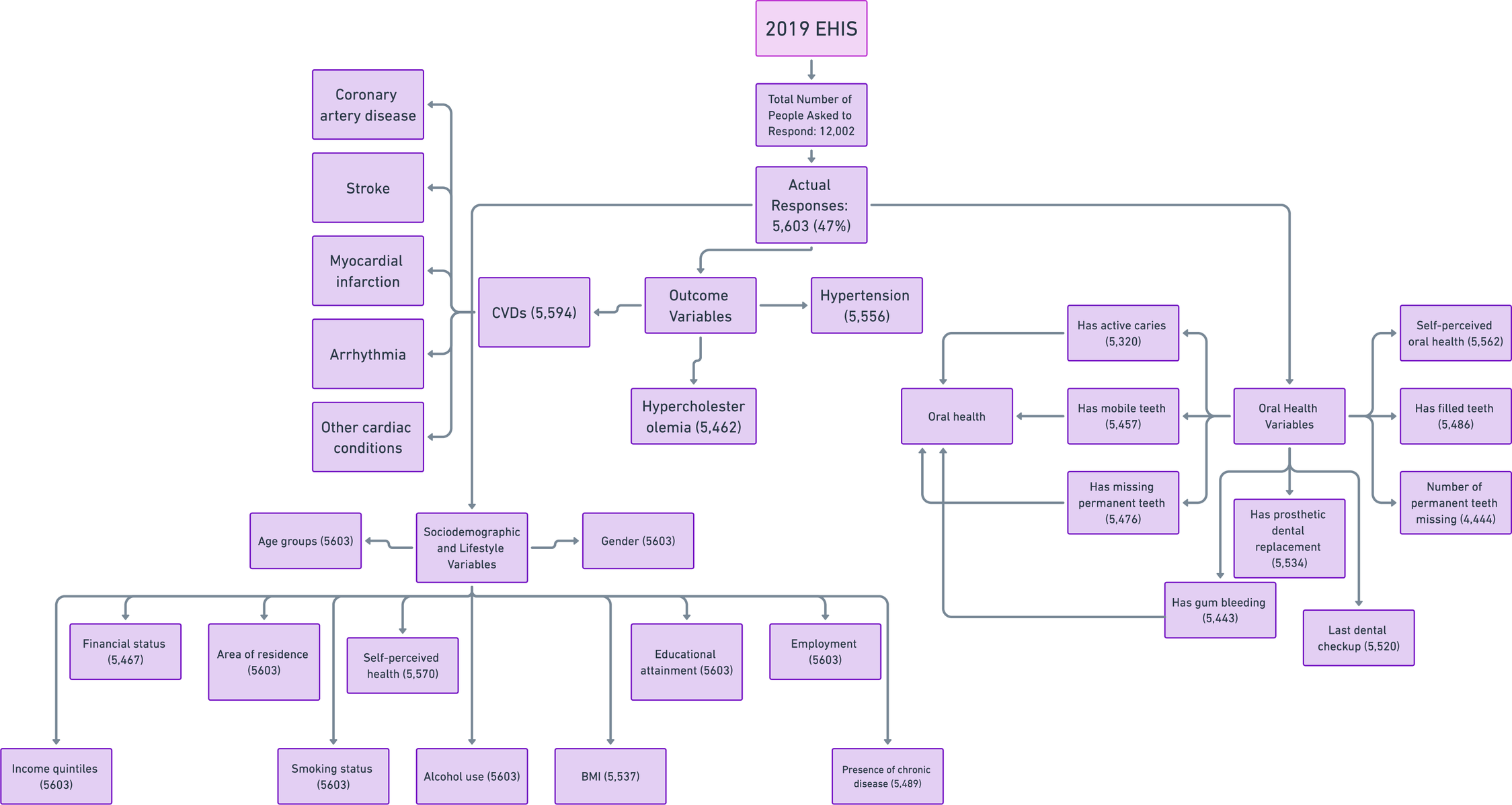

Supplement: S1 Fig — (TIF) [file pone.0301466.s004.tif]
