# Supplementary material for: Role of oral health in heart and vascular health: A population-based study
Source: PLoS One. 2024 Apr 18;19(4):e0301466. doi: 10.1371/journal.pone.0301466 (PMC11025934; doi:10.1371/journal.pone.0301466)
Supplement: Attachment — Submitted filename: 3 review.docx [file pone.0301466.s005.docx]

Comments from the reviewer:

This study titled ‘Role of oral health in heart and vascular health: A population-based study” aimed to assess the impact of oral health indicators on hypertension, CVDs, and hypercholesterolemia among the Hungarian population.

The findings of the manuscript revealed that suboptimal oral health markers were found to be significantly associated with increased risk for negative cardiac outcomes. Conversely, improvements in oral health parameters are linked to a diminution in the risk profiles of these outcomes.

Overall, the manuscript is clear and very well written and is of importance considering that it fills a gap in the literature on the Central and Eastern European region.
